# Studying exercise-induced affect in older adults: how the circumplex model could enhance theoretical and practical advances in the field

**DOI:** 10.3389/fragi.2026.1780273

**Published:** 2026-06-03

**Authors:** Attila Szabo, Celal Bulgay, Erzsébet Stephens-Sarlós, Róbert Járai, Angéla Somogyi, Szilvia Boros, Robert Podstawski, Ferenc Ihász, Roberto Ruíz-Barquín, Ricardo de la Vega

**Affiliations:** 1 Faculty of Health and Sport Sciences, Széchenyi István University, Győr, Hungary; 2 Faculty of Sports Science, Bingöl University, Bingöl, Türkiye; 3 Department of Physiotherapy, University of Warmia and Mazury, Olsztyn, Poland; 4 Facultad de Formación de Profesorado y Educación, Universidad Autónoma de Madrid, Madrid, Spain; 5 Departamento de Educación Física, Deporte y Motricidad Humana, Universidad Autónoma de Madrid, Madrid, Spain

**Keywords:** aging, arousal, circumplex model, core affect, exercise, physical activity, valence

## Abstract

Although decades of research show the health benefits of regular exercise in older adults, the affective mechanisms underlying these benefits remain poorly understood. In fact, research on exercise-induced affect has generally lacked a clear framework for explaining how specific exercise features influence emotional experiences in older adults. This theoretical paper addresses this critical gap by proposing the circumplex model of affect as a comprehensive approach to understanding how exercise modifies valence (pleasure–displeasure) and arousal (activation–deactivation) in older adults. Using interdisciplinary evidence, we explore how neurobiological mechanisms (e.g., BDNF-mediated neuroplasticity and oxidative stress regulation) and hormonal pathways may differently affect affective valence and arousal, the two main dimensions of the circumplex model, during aging. We also explore how key exercise variables, such as intensity, type, duration, and frequency, are linked to distinct affective profiles within the circumplex model, and how individual differences in cognition, health, and socioeconomic status influence these relationships. Our review of the literature reveals that heavy reliance on one-dimensional mood measures has masked important differences between emotional quality and activation level. We argue that using the circumplex approach allows for a clearer understanding of exercise affect links and moves the field beyond the vague claim that exercise improves mood. This circumplex theoretical framework could provide a stronger foundation for developing targeted, evidence-based exercise interventions to improve affective outcomes alongside physical health benefits in older adults.

## Introduction

Exercise, defined as planned physical activity (Caspersen et al., 1985), offers many well-documented benefits for older adults’ physical health, mental wellbeing, and overall quality of life (Bangsbo et al., 2019). However, an important question remains insufficiently explored. How does exercise systematically influence subjective affective experiences in this population, as conceptualized by the circumplex model of affect (Russell, 1980)? This model conceptualizes emotional experiences within a two-dimensional structure defined by valence (pleasure–displeasure) and arousal (activation–deactivation). In this review, the model is adopted not merely as a descriptive vocabulary but as a theoretical framework. Accordingly, affective responses to exercise are evaluated based on the combined structure of these two dimensions. In this context, studies that explicitly apply the circumplex model are distinguished from those that assess valence and arousal separately. The latter are not considered direct applications of the model but are interpreted as circumplex-inferential evidence. This distinction allows for a clearer understanding of the differences between approaches. Since exercise directly influences arousal (Hogan et al., 2013), the circumplex model might offer a comprehensive framework for examining a wide range of affective responses to exercise in older adults.

Affect, mood, and emotion are related but different psychological concepts. Core affect, situated at the intersection of affective valence and arousal in the circumplex space, reflects a basic, consciously accessible, emotional state controlled by neuropsychophysiological processes that are always available as simple, primitive feelings (Russell, 2003). In contrast, emotions are usually directed at specific objects or events, involving conscious evaluations and physiological responses to internal or external stimuli (Scherer, 2005). Conversely, moods are more pervasive, longer-lasting emotional states lacking a clear object or immediate cause (Russell, 2003). Core affect serves as a constant backdrop to awareness, always present even when not in conscious focus, whereas emotions tend to occur in episodes, and moods persist longer. This distinction is important for exercise research, as physical activity may influence the underlying core affective state by altering both valence and arousal, without necessarily triggering specific emotions or significantly affecting longer-term mood patterns.

While there is agreement that exercise generally boosts mood (Kilpatrick, 2008), gaps remain in understanding how specific exercise parameters (e.g., modality, intensity, duration, and frequency) differently affect the two core aspects of valence and arousal, and thus the core affect, in older adults. Most research reports overall improvements in mental wellbeing or reductions in depressive symptoms (Moraes et al., 2007) without differentiating between changes in emotional valence and arousal levels. This lack of detail hampers the development of exercise programs aimed at affective outcomes, such as fostering calm contentment versus energized excitement in everyday life.

The circumplex model is valuable in studying exercise behavior in older adults, whose affective regulation intersects with age-related physical changes and varied health parameters. Understanding how exercise influences both the quality (valence) and intensity (arousal) of affective experiences can inform personalized interventions that match individual preferences, capabilities, and therapeutic goals. For example, one person may benefit most from low-arousal positive states (relaxation, tranquility). At the same time, another individual might seek high-arousal positive states (vigor, excitement), which are obscured by unidimensional measures of affective states.

The present paper addresses this gap by synthesizing existing evidence on exercise and affect in older adults through the lens of the circumplex model, thereby providing a sound theoretical infrastructure for the more uniform study of exercise-related affect in older adults. We examine physiological mechanisms, explore how exercise characteristics and contextual factors shape affective responses, and identify research needs for developing evidence-based interventions that optimize emotional outcomes. Our goal is to advance from general claims about exercise and affect toward a more precise understanding of how physical activity can be tailored to produce specific affective experiences that enhance quality of life in later adulthood.

The present study follows a narrative (non-systematic) review approach. The literature was identified through searches in major electronic databases (e.g., PubMed, Scopus, and Web of Science), complemented by Google Scholar, which has been shown to cover 98%–100% of scientific journal articles indexed in major databases (Chen, 2010; Gehanno et al., 2013) using relevant keywords related to exercise, physical activity, affect, aging, and the circumplex model. The search was limited to studies published between 01 January 2000, and 31 December 2025, and restricted to articles written in English. Studies were selected based on their relevance to the research topic, with a focus on peer-reviewed articles examining neurobiological, psychological, and affective responses to exercise in older adults. Priority was given to experimental studies, systematic reviews, and meta-analyses where available. Rather than applying a formal systematic review protocol (e.g., PRISMA), this review aims to provide a conceptual synthesis of the existing literature and to integrate findings across disciplines.

## The circumplex model of affect in older adults

### Theoretical framework

**FIGURE 1:**
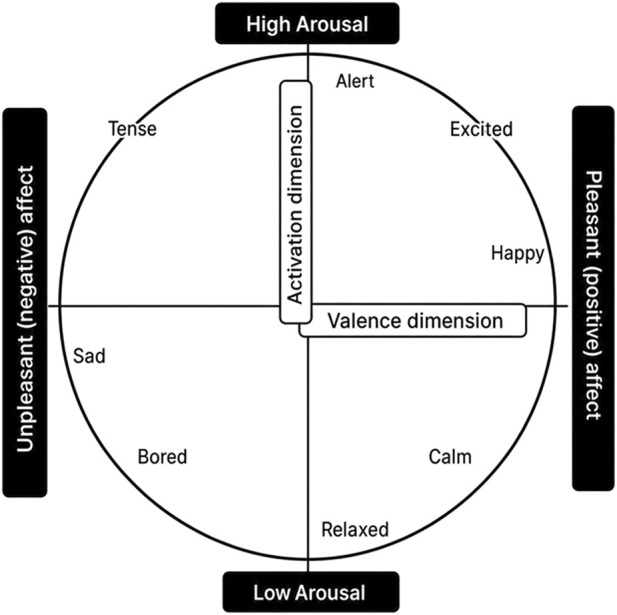
The circumplex model of affect.

The circumplex model (Figure 1) positions core affect within a two-dimensional space defined by two perpendicular axes: valence, which ranges from displeasure to pleasure, and activation, which spans from low to high arousal (Russell, 1980; 2003). In this affective space, specific feeling states are located at points defined by the intersection of pleasantness and activation. For instance, when high arousal coincides with positive valence, people experience excitement or enthusiasm. Conversely, low arousal paired with positive valence mirrors calmness and contentment. The same dimensional framework applies to negative valence states; high arousal combined with displeasure indicates anxiety or distress. On the other hand, low arousal paired with negative valence depicts feelings like lethargy or sadness (Barrett and Bliss-Moreau, 2009).

This framework fosters more precise analysis of affective responses to exercise than unidimensional approaches. Rather than simply asking whether exercise improves mood, we can investigate how different exercise characteristics shift individuals along both dimensions simultaneously. For example, high-intensity interval exercise might increase both arousal and positive valence, producing energized positive states, while gentle stretching might maintain positive valence while decreasing arousal, fostering calm relaxation (Kwan et al., 2017).

### Application to aging populations

Applying the circumplex model to older adults requires consideration of age-related changes in physiological mechanisms regulating affective experience. Research indicates that regular exercise can slow down age-related declines in regulatory systems, including hormonal balance and neurotransmitter function, thereby indirectly supporting affect regulation (Zouhal et al., 2022). Understanding these relationships through the circumplex framework could allow researchers to examine whether exercise interventions differentially influence valence versus arousal dimensions, and whether age-related physiological changes moderate these effects. Individual differences in baseline fitness, mental/cognitive status, and health conditions further complicate the prediction of affective responses, necessitating careful attention to participant characteristics (Johnson et al., 2007; Forster et al., 2021).

## The circumplex and research on exercise-related affect in older adults

An emerging body of research has employed circumplex-compatible measurement approaches to examine exercise-induced affective responses in older adults, typically using indicators of affective valence and arousal (Table 1). Despite the growing use of such measures, the explicit application of the circumplex model remains limited. Recent methodological evidence demonstrates that the Feeling Scale (FS; Hardy and Rejeski, 1989) and the Felt Arousal Scale (FAS; Svebak and Murgatroyd, 1985) are reliable and valid for use in older populations (Sobrinho et al., 2025), providing a solid psychometric foundation for circumplex-informed research. Their brevity and ease of administration further support their suitability for repeated and ecologically valid assessment in aging samples.

**TABLE 1:** Published research examining affective responses to exercise in older adults using the circumplex model or circumplex-inferential approaches.

Author and year	Participants (age, N)	Type of exercise	Intervention length/Study duration	Measures/Instruments	Major findings
Studies using the circumplex model					
de Souza et al. (2025)	Older women; 60–75 years; N = 15	Multicomponent training, resistance training, walking	Acute crossover; three single sessions (∼40 min each)	FS, FAS, PACES, PRETIE-Q	Explicit circumplex interpretation; all exercise modes elicited positive affective responses, with movement toward pleasant–activated states. Multicomponent training best maintains positive valence
Follador et al. (2019)	Elderly women; Tai Chi (n = 26), yoga (n = 25), stretching (n = 19); N = 70	Tai Chi, yoga, and stretching classes	Acute; single session per modality	FS, FAS, RPE	Explicitly used the circumplex model; affective valence and arousal shifted from low-activation pleasure (baseline) to high-activation pleasure quadrant across all three activities. Exercise perceived as pleasant with moderate exertion
Ribeiro et al. (2021)	Older women; ≥60 years; N = 46 completers	Resistance training (four exercise orders)	12-week supervised program; 3 sessions/week	FS, FAS	Findings explicitly interpreted within the circumplex model; participants consistently occupied pleasant–activated affective states pre- and post-training
Studies using a circumplex-inferential approach					
Benites et al. (2016)	Older women; N = 24	Resistance training at prescribed intensity	8 weeks; 3 sessions/week	FS, RPE	Perceived exertion and pleasure were assessed over an 8-week resistance training program; pleasure responses remained positive across the intervention period, consistent with the valence dimension of the circumplex
Elsangedy et al. (2021)	Physically inactive older women (mean age 66 years; N = 32)	Self-selected resistance training	12 weeks	FS, FAS, RPE	Training sessions perceived as pleasant with low-to-moderate exertion; affective responses consistent with pleasant–activated states
Hogan et al. (2013)	Older adults subsample (19–93 years overall; older N = 144)	Moderate-intensity cycling	Acute: 15 min	Modified circumplex-based emotion sampler	Exercise increased high-arousal positive affect across ages; older adults maintained low-arousal positive affect post-exercise, consistent with circumplex predictions
Orsano et al. (2018)	Elderly hypertensive women; 67.1 ± 6.9 years; N = 15	Traditional RT vs. high-velocity RT	Acute crossover; single sessions	FS, RPE	Both traditional and high-velocity resistance training elicited positive affective responses, with no differences between protocols; the results are inferentially aligned with the pleasant quadrants of the circumplex
Pereira et al. (2022)	Older women; 66.1 ± 3.9 years; N = 16	Resistance training to momentary muscle failure	Acute crossover; single sessions	FS, sRPE	Moderate loads elicited greater pleasure and lower perceived exertion; these findings map inferentially onto the valence dimension of the circumplex
Richardson et al. (2018)	Older adults; 60–79 years; N = 10	Resistance training (HVLL vs. LVHL)	Acute sessions	FS, FAS, RPE	Perceptual responses to different velocity/load conditions assessed; both conditions elicited positive affective states, with findings inferentially consistent with circumplex quadrants
Richardson et al. (2020)	Older adults; N = 10	Resistance training (HVLL vs. LVHL)	Acute sessions	FS, FAS, PAAS, RPE, VAS, PACES	Both conditions elicited positive affective responses; the findings align with the pleasant–activated quadrants but lack explicit circumplex framing
Richardson et al. (2020)	Older adults; 60–79 years; N = 40	Resistance training (HVLL and LVHL, once or twice weekly)	10-week intervention	FS, FAS, PAAS, RPE, VAS, PACES	Positive affect and enjoyment were maintained across conditions despite differences in fatigue; results are inferentially consistent with circumplex dimensions

Studies are ordered with those explicitly framing or interpreting results within the circumplex model of affect listed first, followed by studies that inferentially align affective responses with circumplex dimensions (valence and arousal) without explicit theoretical framing. Entries marked with an asterisk (*) are additions identified through a supplementary literature search. FS, feeling scale; FAS, felt arousal scale; PAAS, physical activity affect scale; RPE, rating of perceived exertion; sRPE, session rating of perceived exertion; VAS, visual analog scale; PACES, physical activity enjoyment scale; HVLL, High-Velocity Low-Load; LVHL, Low-Velocity High-Load; RT, resistance training; PRETIE-Q, preference for and tolerance of the intensity of exercise questionnaire.

### Direct circumplex focus and discussion

Only a small number of intervention studies have explicitly framed and interpreted affective responses to exercise in older adults within the circumplex model. Notably, de Souza et al. (2025) examined acute affective responses to multicomponent training, resistance training, and walking in older women and interpreted FS and FAS responses directly within the circumplex space. Across all modalities, exercise elicited affective states characterized by positive valence and moderate-to-high activation, with multicomponent training showing particular advantages in maintaining positive valence throughout the session.

Similarly, Ribeiro et al. (2021) explicitly adopted the circumplex framework to interpret affective responses to a 12-week resistance training program in older women. Using repeated FS and FAS assessments, the authors reported that participants consistently occupied an activated pleasant affective state both before and after exercise sessions, suggesting that regular resistance training may support a stable, positively valence-characterized affective profile over time. These two studies represent focused empirical applications of the circumplex model to exercise-related affect in older adults.

### Indirect circumplex focus and discussion

A greater number of studies have assessed affective responses to exercise in older adults using circumplex-compatible measures. However, they interpreted their findings without explicitly referencing the circumplex model. For example, Richardson et al. (2018); Richardson et al. (2020) compared affective responses across different resistance training protocols. They found that high-velocity, low-load exercise elicited slightly more favorable valence and arousal profiles than low-velocity, high-load protocols. Although FS and FAS responses were analyzed separately, the results indicate that exercise modality and intensity differentially influence both affective dimensions in ways consistent with circumplex assumptions.

Walking-based research has also identified affective trajectories consistent with the circumplex framework. Ekkekakis et al. (2008) demonstrated that brief walking sessions increased activation and positive valence during exercise, followed by shifts toward pleasant deactivation during recovery in middle-aged and older adults. This pattern, marked by movement mainly along the arousal dimension with relative stability of valence, aligns with circumplex models, even when not explicitly mapped into two-dimensional affective space. However, this study did not specifically focus on older adults. Similarly, the aquafitness study by Ábel et al. (2023), which showed a positive shift in core affect following aquafitness from a pre-existing positive baseline in middle-aged women. Using a different exercise modality and a circumplex-based emotion sampler, Hogan et al. (2013) demonstrated that moderate-intensity cycling increased high-arousal positive affect across age groups. In contrast, older adults uniquely maintained low-arousal positive affect after exercise, a pattern consistent with circumplex-based interpretations of age-related differences in affect regulation.

Resistance exercise studies also support a circumplex-inferential analysis of affective responses in older adults. Using self-selected resistance training, Elsangedy et al. (2021), found that physically inactive older women consistently perceived exercise sessions as pleasant and of low-to-moderate exertion across a 12-week exercise intervention, suggesting stable affective responses within a pleasant–activated region. Similarly, Pereira et al. (2022) demonstrated that moderate resistance loads performed to momentary muscle failure elicited greater pleasure and lower perceived exertion than heavy or light loads in older women. Although neither study explicitly framed findings within the circumplex model, both indicate that exercise prescription variables, particularly load selection and perceived effort, systematically influence affective valence and, by implication, positioning within circumplex space.

Finally, ecological momentary assessment (EMA) studies highlight greater variability in affect–exercise relationships in daily life. Kanning et al. (2015) and Kanning and Hansen (2017) reported that physical activity in adults aged 50 and older was reliably associated with increased energetic arousal and reduced calmness. In contrast, changes in valence depended on contextual and motivational factors such as psychological need satisfaction. Similarly, Hevel et al. (2021) showed that momentary energy predicted subsequent physical activity, and Hudgins and Maher (2025) revealed that habit strength moderated associations between movement behaviors and affective activation. Although these naturalistic studies do not explicitly employ the circumplex model, their findings support the notion that valence and arousal can vary independently and are shaped by contextual and individual differences. Interpreting such results within a circumplex framework may therefore enhance theoretical integration, comparability across study designs, and identification of distinct affective response patterns to exercise in older adults.

## Physiological mechanisms linking exercise to circumplex affect

### Neurobiological pathways

Exercise influences affective states via multiple neurobiological pathways relevant to both dimensions of the circumplex model. Physical activity can boost brain-derived neurotrophic factor (BDNF) expression and support hippocampal neurogenesis, processes that may underlie improvements in affective regulation (Erickson et al., 2011; Håkansson et al., 2016). The connection between adult hippocampal neurogenesis and affective disorders suggests that exercise-induced neuroplasticity contributes to sustained improvements in affective valence (Gomes-Leal, 2021). Additionally, exercise regulates inhibitory control and executive function, potentially enhancing the capacity to regulate arousal levels in response to environmental demands (Peruyero et al., 2017).

**FIGURE 2:**
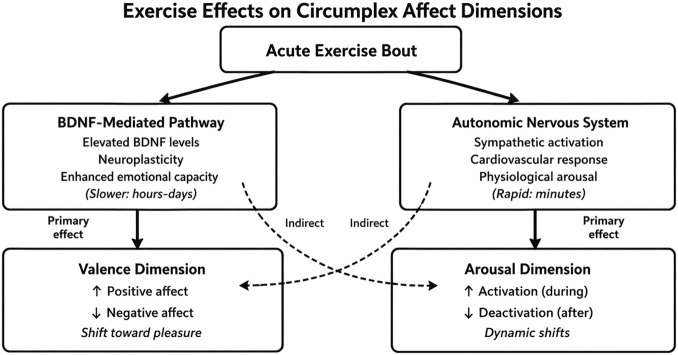
The effect of brain-derived neurotrophic factor (BDNF) on circumplex dimensions.

The effects on BDNF (Figure 2) appear particularly robust following acute exercise bouts, with research documenting elevated levels following 35-min sessions combining exercise, cognitive training, and mindfulness (Håkansson et al., 2016). These neurobiological changes may manifest differently across the circumplex quadrants (Stillman et al., 2016). For example, BDNF-mediated neuroplasticity might primarily influence valence by reducing negative affect and enhancing positive emotional capacity. In contrast, arousal responses depend more directly on activation of the autonomic nervous system during and after exercise.

### Oxidative stress and antioxidant responses

**FIGURE 3:**
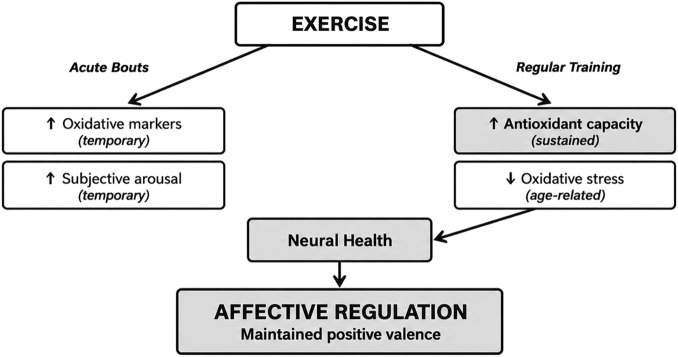
Conceptual model linking exercise, oxidative stress, and affective regulation in older adults. Acute exercise is associated with transient increases in oxidative markers and subjective arousal, whereas regular exercise training is proposed to enhance endogenous antioxidant capacity and attenuate age-related oxidative stress. Reduced oxidative stress may support neural health and contribute to more stable affective regulation, including the maintenance of positive valence over time. This model represents a theoretical synthesis of findings from exercise physiology and affective neuroscience rather than a fully specified causal pathway.

Exercise enhances antioxidant responses and reduces oxidative stress, supporting neural health and potentially influencing affective regulation in older adults (El Assar et al., 2022; Radak et al., 2008; Simioni et al., 2018). These effects appear particularly relevant for maintaining positive valence over time, as oxidative damage accumulates with age and contributes to declines in mood and cognitive function (Liguori et al., 2018). Regular exercise training may lead to sustained improvements in antioxidant capacity that support baseline affective states, whereas acute exercise bouts produce temporary shifts in both oxidative markers and subjective arousal (Figure 3).

### Hormonal regulation

Exercise modulates anabolic and catabolic hormone levels, with systemic effects that develop with aging (Hayes et al., 2017; Kraemer and Ratamess, 2005; Zouhal et al., 2022). These hormonal changes likely contribute to mood alterations through multiple pathways, including regulation of the stress response and maintenance of metabolic balance. The link between hormonal shifts and circumplex dimensions remains poorly studied. However, it seems likely that hormonal adaptations primarily influence valence through stress-system regulation, whereas their impact on arousal might vary with exercise timing and physical effort intensity.

## Exercise parameters and circumplex outcomes

Since the circumplex model focuses on affect rather than exercise, it cannot directly determine exercise parameters. Instead, its value lies in mapping existing exercise variables onto the two affective dimensions (i.e., valence and arousal) to create testable predictions about how specific exercise traits might differentially influence core affect within the circumplex space in older adults.

### Intensity and arousal modulation

Exercise intensity is considered an important, though not the sole, determinant of arousal levels within the circumplex model. However, this relationship is unlikely to be linear in older adults due to the positivity bias, age-related changes in emotion regulation processes, and reduced physiological arousal levels (Nigam and Kar, 2025). High-intensity exercise naturally elevates physiological arousal through cardiovascular and metabolic demands, potentially translating to increased subjective activation (Lucas et al., 2015). Nevertheless, the assumption of a direct and uniformly positive association between intensity and subjective arousal should be interpreted with caution. The relationship between objective physiological arousal and subjective affective arousal remains poorly understood, particularly regarding how these relationships change with training adaptations and individual differences in fitness. For instance, EMA studies with older adults show that objectively measured physical activity levels (via accelerometry) correspond with increased energetic arousal but do not consistently predict improved affective valence, while body mass index and psychological need satisfaction might moderate these relationships (Kanning et al., 2015; Kanning and Hansen, 2017). Accordingly, the present manuscript adopts a more cautious, non-causal interpretation of the intensity–arousal relationship, acknowledging potential moderating factors and population-specific variability.

Moderate-intensity continuous exercise may represent a feasible and potentially beneficial option for many older adults, as it balances physiological demands with sustainable participation. However, because high-intensity exercise is not feasible for a large proportion of aging individuals, the influence of exercise intensity on affective dimensions such as arousal and valence is likely to vary across individuals rather than follow a uniform pattern.

While regular moderate-intensity exercise is associated with well-established cardiovascular adaptations (Nystoriak and Bhatnagar, 2018), the mechanisms through which these physiological changes translate into affective outcomes remain insufficiently understood. In this context, determining appropriate exercise intensity in older adults may be better informed by individual fitness history, health condition, and personal motivation, which reflects long-term exposure and adaptation to physical activity, rather than relying solely on general factors such as personality traits or momentary affective states. Accordingly, the relationship between exercise intensity and affective responses should be considered as individualized and context-dependent, rather than universally determined.

### Modality and affective quality

Different types of exercise can produce distinct affective profiles within the circumplex space. Aerobic activity, resistance training, and flexibility-based practices combined with breathing exercises (e.g., hatha yoga) differ in their physiological demands and patterns of cognitive engagement, which may influence valence and arousal in different ways (Stevens et al., 2016; Evmenenko and Teixeira, 2022; Henriques et al., 2023). While these findings suggest that exercise modality influences affective responses, the available evidence remains limited and heterogeneous. Moreover, much of the existing research, including large-scale cross-sectional studies (Chekroud et al., 2018), does not permit causal inferences. Therefore, the relationship between exercise modality and affective outcomes should be interpreted with caution, and further controlled and longitudinal studies are needed to clarify these associations.

Cross-sectional data involving over 1.2 million individuals suggest that different activity types are associated with distinct mental health outcomes (Chekroud et al., 2018). However, translating these associations into predictions about circumplex positioning requires controlled experimental work that examines how specific modalities shift valence and arousal within individuals over time. The interaction between modality and individual characteristics likely determines which activities produce the most favorable affective profiles for each person.

### Duration and frequency considerations

Both exercise duration and frequency may influence affective valence and activation, as well as longer-term baseline affect; however, these relationships should be interpreted with caution. Single exercise bouts are often associated with immediate shifts in circumplex position that gradually diminish over time (Szabo and Kovacsik, 2019), whereas regular participation may be linked to more stable affective patterns. This distinction between acute and longer-term associations is illustrated by Ábel et al. (2023), who reported increased core affect following a single session of aquatic exercise in middle-aged women; however, participants already exhibited positive baseline valence, suggesting that such responses may partly reflect prior engagement in regular activity. Similarly, (Richardson et al., 2020), observed maintained positive affective responses across different training frequencies in older adults, although these findings do not allow clear attribution to exercise frequency alone. Likewise, Pereira et al. (2022) reported pleasant and activated affective states following a 12-week resistance training program, but these outcomes may also be influenced by broader contextual factors associated with structured and engaging activities. Therefore, the effects of exercise duration and frequency on valence and arousal are better understood as context-dependent and potentially interacting with other behavioral and environmental influences, rather than as isolated causal determinants. Distinguishing immediate post-exercise responses from more sustained affective patterns remains essential for interpreting these relationships.

## Moderating factors and individual differences

### Cognition and health status

Baseline cognitive abilities and health conditions substantially moderate affective responses to exercise. Research demonstrates that exercise interventions can improve cognitive function in healthy older adults (Carta et al., 2021), suggesting cognitive and affective changes may occur jointly. However, the direction of causality remains unclear; improved affect may facilitate cognitive engagement, enhanced cognition may support better affect regulation, or both may reflect shared underlying mechanisms, such as improved neural health.

Functional capacity and chronic disease burden create additional complexity. Exercise is considered a non-pharmacological intervention for several age-related conditions (Izquierdo et al., 2021), with disease management possibly mediating affective improvements. Individuals with greater functional limitations may experience different circumplex trajectories as exercise improves physical capabilities and reduces disease symptoms alongside direct affective effects.

### Socioeconomic and demographic factors

Socioeconomic status may influence engagement in structured exercise, although this relationship depends on how physical activity is defined and measured. While some evidence suggests that individuals in higher socioeconomic groups report greater participation in planned and leisure-time exercise (McPhee et al., 2016). This does not necessarily imply lower overall physical activity among lower socioeconomic groups. Individuals in lower socioeconomic strata may engage in higher levels of occupational or daily physical labor, which may differ in structure, purpose, and affective experience from leisure-based exercise. Gender differences in exercise responses and nutritional needs further complicate intervention design, particularly regarding hormonal influences in older women (Mattioli et al., 2022).

These distinctions are important when considering affective outcomes, as access to structured exercise environments, perceived autonomy, and activity context may shape both participation and emotional responses. Barriers such as limited transportation, reduced access to exercise facilities, and lower social support can differentially influence opportunities for structured exercise across socioeconomic groups (Rai et al., 2020). At the same time, the types of physical activity available in different socioeconomic contexts, whether leisure-based or work-related, may lead to distinct affective experiences. Therefore, rather than assuming a simple association between socioeconomic status and physical activity levels, it is more appropriate to consider how the type, context, and meaning of activity interact with socioeconomic conditions to influence affective responses. Addressing equity in exercise interventions requires acknowledging these distinctions and designing approaches that account for diverse forms of physical activity and resource constraints.

### Environmental and social context

Exercise settings can also substantially influence affective responses through both physical environmental features and social dynamics. Natural outdoor environments may offer greater affective benefits than indoor facilities, with research suggesting that outdoor exercise yields favorable psychological outcomes (Calogiuri et al., 2015). The mechanisms underlying these effects may involve multiple pathways, including aesthetic experiences, exposure to natural light, good air quality, and psychological restoration processes, all of which may contribute to enhanced valence and optimal arousal regulation during outdoor exercise (Wicks et al., 2022).

Social contexts are also instrumental in shaping exercise-induced affective experiences. Group-based exercises provide opportunities for social interaction alongside physical activity, with social engagement representing a well-established determinant of older adults’ wellbeing (Tcymbal et al., 2022). Interventions that promote parallel social interaction and exercise may yield synergistic affective benefits. However, distinguishing social from exercise effects needs careful experimental research design. Individual versus group settings likely produce different circumplex profiles, with group activities potentially increasing arousal via social stimulation, whereas individual exercise allows for more flexible modulation of arousal.

## Digital health interventions and personalization

### Technology-mediated exercise delivery

Digital health technologies offer potential opportunities for personalized exercise prescription and real-time affective monitoring; however, their applicability in older adults requires careful consideration. Platforms that deliver individualized exercise programs may adapt to users’ capabilities and preferences, potentially supporting affective outcomes (Sun et al., 2021). Nevertheless, age-related cognitive and motor declines may limit usability and sustained engagement, even in individuals with adequate technology acceptance and digital literacy. As such, the effectiveness of these approaches is likely to vary across individuals.

Exergames and interactive digital programs may provide engaging alternatives to traditional exercise for some users, and some evidence suggests they can improve physical and cognitive outcomes while maintaining engagement (Gallou-Guyot et al., 2023). However, these technologies may also introduce cognitive or usability challenges that could negatively influence the emotional experience, particularly in older populations.

Wearable digital technologies allow continuous monitoring of physiological parameters during exercise and may facilitate examination of the relationship between objective physiological responses and subjective affective states (Lee et al., 2021). Combining biometric data with real-time affect assessments could provide insights into how exercise parameters relate to movement within the circumplex space. However, the interpretation, accessibility, and meaningful use of such data may differ substantially across individuals. Therefore, rather than assuming uniformly positive effects, the role of digital technologies in exercise contexts should be considered context-dependent, with the potential for both beneficial and adverse affective outcomes. Careful attention to usability, individual differences, and age-related limitations is essential when integrating these technologies into exercise interventions for older adults.

### Digital literacy and accessibility

Technology acceptance among older adults depends substantially on digital literacy, perceived usability, and prior experience with digital tools (Schroeder et al., 2023). The rapid expansion of telehealth and digital exercise platforms presents both opportunities and challenges for reaching older populations through technology (Ho and Merchant, 2022) Designing effective digital interventions requires a thorough understanding of adoption barriers while providing adequate training and support to sustain engagement.

Integrating digital literacy training into exercise programs can encourage the adoption of technology and sustained participation (Kebede et al., 2022). Co-design methods that involve older adults in developing digital health solutions enhance their relevance and usability, thus ensuring that interventions meet actual needs rather than assumed ones (Mannheim et al., 2019). The emotional experience of using digital exercise platforms can affect both immediate reactions and long-term engagement. Technology that is frustrating or cognitively demanding might reduce positive feelings or increase negative ones in the circumplex space. Therefore, equity considerations are essential. Digital interventions could widen existing disparities if they are not accessible to those with limited resources or technological skills.

### Ethical and privacy considerations

Digital health interventions raise significant ethical concerns related to data privacy, informed consent, and equitable access (Finco et al., 2024). Gathering affective and physiological data poses privacy risks that require robust protections. Making sure that older adults understand how their data will be used and stored is a key ethical responsibility. Additionally, issues like the digital divide may prevent vulnerable older adults from accessing technology-based interventions unless deliberate efforts are made to ensure universal accessibility, regardless of technological literacy or financial resources.

## Critical research gaps and methodological needs

### Direct investigation of circumplex dimensions

The most critical research gap is the shortage of studies that explicitly examine the effects of exercise on both the valence and arousal dimensions in older adults. While substantial evidence supports general affective benefits, few investigations systematically assess how exercise parameters shift individuals’ core affect within the circumplex space. This gap reflects broader limitations in affective assessment, as many studies rely on unidimensional measures of affect that cannot distinguish changes in valence from those in arousal.

Future research should employ psychometric tools specifically designed to assess circumplex dimensions, like the FS and the FAS. This method enables a more precise mapping of affective trajectories before, during, and after exercise. Accordingly, future studies could clarify which exercise parameters preferentially influence valence versus arousal, how these effects vary across individuals, and whether certain parameter combinations produce optimal affective outcomes. Longitudinal research designs that track circumplex position over extended interventions could reveal how acute affective responses translate into sustained, long-lasting baseline improvements.

### Mechanistic understanding

Understanding the physiological and psychological mechanisms linking exercise to specific circumplex outcomes remains incomplete. While evidence reveals neurobiological changes, hormonal adaptations, and cognitive improvements, how these mechanisms translate into shifts in valence and arousal remains to be elucidated. Research integrating biomarkers with repeated affective assessments could illuminate these pathways, potentially identifying individual differences in the activation of mechanisms that explain variable affective responses.

Recent approaches combining biometric sensors with ecological momentary assessment of affect offer promising tools to examine real-time relationships between physiological responses and subjective experiences (Benedyk et al., 2024). Importantly, such methods are not intended to categorize responses into discrete emotions, but rather to capture continuous variations in affective states that can be interpreted within the circumplex structure. These approaches may help explain individual differences in affect–physiology coupling and why similar exercise stimuli lead to different affective outcomes.

Accordingly, integrating physiological and affective data may support a more nuanced understanding of how exercise influences positioning within the valence–arousal space, ultimately informing more individualized and context-sensitive exercise interventions.

### Long-term sustainability and adherence

While acute affective responses to exercise have received some attention, how these immediate effects relate to long-term participation and sustained affective benefits is still unclear, particularly among older adults. Investigating whether positive acute affective responses predict better adherence in this population could provide valuable insights for intervention design, highlighting the importance of affective experiences alongside physiological outcomes. Furthermore, examining how baseline affective states shape both the initiation and maintenance of exercise behavior would help clarify the bidirectional relationship between affect and exercise. Additionally, future research should examine whether the affective benefits of exercise interventions vary across different stages of later life, because progressive cognitive, sensory, and physical declines in advanced old age may alter both the capacity to engage in structured exercise and the resulting affective responses (Martínez-Velilla et al., 2019). Understanding these age-related boundaries is essential for determining the extent to which exercise-based affective interventions remain viable across the aging continuum.

## Future directions for research and practice

### Personalized exercise prescription

Moving toward truly personalized exercise interventions requires understanding individual characteristics including personality, preferences, baseline affect, cognitive status, and health conditions moderate affective responses to different exercise parameters. Rather than seeking universal optimal exercise prescriptions, research should identify decision rules for matching individuals to exercise characteristics likely to produce favorable circumplex outcomes for their specific profile and goals.

Developing such personalization algorithms requires large-scale studies that collect detailed individual characteristics data alongside varied exercise exposures and repeated affective assessments. Machine learning approaches might identify complex interaction patterns that determine optimal exercise prescriptions, though interpretability remains essential for clinical and practical applications. The ultimate goal is to create evidence-based tools that enable practitioners to design exercise programs targeting specific affective outcomes while accounting for individual variation.

### Integration across disciplines

Advancing understanding of exercise-affect relationships in older adults requires integrating multiple disciplines. Exercise physiology offers essential knowledge about bodily responses, psychology sheds light on affective experiences and regulation, neuroscience uncovers mechanistic pathways, gerontology adds developmental perspectives, and public health frames population-level concerns. Combining insights from these areas allows for a comprehensive understanding that goes beyond any single discipline (John et al., 2020).

This integration is crucial for older adults, in whom physical activity behaviors reflect complex interactions among biological aging, psychological factors, social circumstances, and environmental contexts. Research adopting ecological frameworks that consider multiple levels of influence yields a richer understanding than approaches that narrow their focus to individual-level factors (Noh and Kim, 2022). Such comprehensive perspectives support the development of interventions that simultaneously address barriers and facilitators across multiple domains.

### Policy and public health implications

Translating research findings into population health benefits requires attention to policy and structural factors. Given that socioeconomic disparities substantially affect participation in physical activity, public health interventions must address accessibility barriers while optimizing emotional outcomes within available resources. This includes ensuring that older adults across socioeconomic strata can access facilities, programs, and technologies that support physical activity engagement.

Developing inclusive, accessible programs requires community engagement and participatory design to ensure interventions meet diverse needs and preferences (Meredith et al., 2023). Policy initiatives should promote equitable access while supporting innovation in delivery methods, including digital health solutions that overcome traditional participation barriers. However, technology-based approaches must include provisions for digital literacy support and avoid exacerbating existing inequities by focusing exclusively on digitally connected populations.

## Conclusion

The circumplex model could provide a valuable theoretical and applied framework for understanding how exercise impacts affective experiences in older adults through the fundamental dimensions of valence and arousal. While substantial evidence supports general mood benefits from physical activity, systematic investigation of how specific exercise parameters produce distinct circumplex profiles remains limited. Addressing this gap requires research that explicitly examines both affective dimensions, employs appropriate psychometric tools, and investigates the physiological and psychological mechanisms linking exercise characteristics to specific affective outcomes.

Multiple physiological pathways, including neurobiological changes, reductions in oxidative stress, and hormonal adaptations, likely contribute to exercise-induced improvements in affect. However, their specific roles in producing valence versus arousal changes require further elucidation. Exercise parameters such as intensity, modality, duration, and frequency presumably influence these dimensions differentially, with individual characteristics and contextual factors substantially moderating responses. Digital health technologies offer promising avenues for personalization and real-time affective monitoring, though concerns about equity and accessibility call for careful attention.

Moving forward requires integrated research approaches that combine biological, psychological, and social perspectives while maintaining a focus on individual variability in affective responses. Large-scale studies that collect detailed participant characteristics alongside varied exercise exposures and repeated circumplex assessments could reveal patterns that enable truly personalized exercise prescription. Such knowledge would support the development of evidence-based interventions that optimize both physical health and emotional wellbeing, ultimately enhancing the quality of life for older adults by strategically leveraging exercise’s affective benefits. The circumplex model could provide a uniform framework for comparing research outcomes.

The long-term goal is to create accessible, equitable exercise programs that account for diverse needs, preferences, and circumstances while systematically targeting positive affective outcomes. Achieving this goal requires sustained research efforts on comparable theoretical grounds, multidisciplinary collaboration, and policy initiatives to ensure that all older adults can access exercise opportunities that enhance their emotional wellbeing. The circumplex model could be used in evaluating the success of such initiatives.
